# Madelung’s disease -a case series from a single-center experience

**DOI:** 10.3389/fsurg.2025.1636822

**Published:** 2025-10-02

**Authors:** Monika Łącka, Julia Wojciechowska, Paulina Bernecka, Hanna Szóstek, Amelia Stolp, Zuzanna Zieniewicz, Martyna Miller, Brygida Ossowska, Jerzy Jankau

**Affiliations:** 1 Plastic Surgery Department, Medical University of Gdańsk, Gdansk, Poland; 2 Student Scientific Association of Plastic Surgery, Medical University of Gdańsk, Faculty of Medicine, Gdansk, Poland; 3 Division of Embryology, Department of Anatomy, Medical University of Gdańsk, Gdansk, Poland

**Keywords:** Madelung's disease, Madelung's disease treatment, multiple symmetric lipomatosis, surgical treatment, Klein's solution, rare metabolic disorder

## Abstract

**Introduction:**

Madelung's disease (MD), also known as multiple symmetric lipomatosis, is a rare metabolic disorder characterized by the accumulation of unencapsulated adipose tissue, predominantly in the head, neck, and upper trunk. Non-surgical treatment options remain limited in effectiveness, making surgical excision the primary therapeutic approach. However, challenges such as intraoperative bleeding and postoperative recurrence necessitate ongoing refinement of surgical techniques.

**Methods:**

We conducted a retrospective case series involving six patients diagnosed with Madelung's disease and treated surgically between 2018 and 2024 at the Department of Plastic Surgery, University Clinical Hospital in Gdańsk. All patients underwent staged surgical excision of pathological adipose tissue following infiltration with Klein's solution to minimize bleeding. Demographic and clinical data, comorbidities, fat distribution patterns (Type I or II), and body mass index (BMI) were recorded. The primary outcome was recurrence in the operated regions over a one-year follow-up period; the secondary outcome was the occurrence of surgical complications.

**Results:**

The study included five male and one female patient, with a mean age of 57 years (range: 44–67). Risk factors included smoking (*n* = 4), alcohol abuse (*n* = 2), and metabolic or systemic comorbidities. The BMI ranged from 21 to 33. All patients underwent successful surgical resection, with no recurrence of adipose tissue in the treated areas during follow-up. Histopathological evaluation confirmed the presence of benign lipomas in all specimens. No major complications, such as excessive bleeding or postoperative infections, were observed.

**Discussion:**

Surgical excision with prior infiltration of Klein's solution appears to be a safe and effective treatment for Madelung's disease, offering low recurrence and complication rates. The use of staged procedures and careful intraoperative management is critical in addressing the disease's vascular and infiltrative nature. While these findings are promising, larger prospective studies are needed to validate the efficacy of this approach and to further optimize surgical strategies for this rare condition.

## Introduction

Madelung's disease (MD), also referred to as multiple symmetric lipomatosis or Launois-Bensaude syndrome, is a rare metabolic condition (1). MD is marked by the progressive and symmetrical accumulation of pathological adipose tissue (2). The disease primarily manifests through the presence of unencapsulated lipomas typically located around the neck, shoulders, and chest (3). The head and neck are the most frequently affected regions. The pathological tissue usually is redistributed symmetrically, forming a “horse collar” appearance of the neck (4). Slow-growing, painless soft tissue containing nonencapsulated masses may extend into both superficial and deep fascial spaces (5).

Madelung's disease was first described by Benjamin Collins Brodie, a British physiologist and surgeon, in 1846. The condition was later systematically summarized and defined as a distinct disease entity by Otto Wilhelm Madelung in 1888 (6, 7).

MD affects approximately 1 in 25,000 people, predominantly occurs in men with a male to female ratio ranging from 15:1 to 30:1 and typically manifests between the ages of 45 and 65 (4, 8, 9). Key risk factors include chronic alcohol consumption (89.5% of cases), smoking (53%), but it can also be associated with several comorbidities such as hyperuricemia, hypertension, obesity, hypothyroidism, diabetes, hyperlipidemia, neurological disorders, and liver conditions, particularly alcoholic liver disease (9). However, these are not always decisive, since cases without such risk factors have been reported. In the study of 54 Chinese patients, almost all were men (mean age 57 years), with alcohol and smoking history in the majority (10). Surgical treatment was performed in 70% of cases, with recurrence observed in 39.5%. Similarly, Pinto et al. (11) reported 17.8% complications such as seromas, hematomas, and infections, with recurrence in 39% of patients. Lipectomy is still considered effective, particularly for functional and aesthetic improvement, but recurrence rates remain high. Lifestyle modifications, including alcohol and tobacco cessation and weight reduction, are strongly recommended to improve outcomes. In research by Li et al. (10) the largest percentage 81.48% were patients with endocrine disease. Malignant tumors occurred in 20.37% of the study participants and the most often affecting the digestive system.

The exact cause of MD remains unclear, although several mechanisms have been proposed. The prevailing hypothesis implicates disturbances in lipolytic pathways and alterations in mitochondrial DNA in the pathogenesis of the disease (9). MD shows a strong correlation with alcohol consumption. Alcohol disrupts mitochondrial function, leading to premature oxidation and point mutations in mitochondrial DNA, particularly in the tRNA-lysine gene (12). In addition, alcohol and other toxins may activate cytochrome P450 enzymes in adipose tissue, resulting in adipocyte apoptosis and inflammation (13, 14). Mitochondrial dysfunction in brown adipose tissue and impaired lipolysis have also been suggested as contributing factors (15).

MD is often associated with metabolic conditions such as hepatic steatosis, hypothyroidism, diabetes, and hypertension (16). Furthermore, Madelung adipose tissue-derived stem cells (MD-ASC) exhibit minor phenotypic and functional differences compared with healthy adipose-derived mesenchymal stem cells (AD-MSC). MD-ASC likely alter the growth and cell surface phenotype of AD-MSC, resulting in possible spread of pathology to healthy tissue (17). Another theory suggests that increased miRNAs may promote adipogenesis by inhibiting the RhoA/ROCK/ERK1/2 pathway (18). However, the exact etiological mechanisms of MD are still under investigation.

MD can be classified according to the anatomical distribution of adipose tissue. Two main classification systems are commonly cited in the literature: the original system proposed by Enzi and the more recent modification introduced by Donhauser et al. Enzi's classification distinguishes two major types of MD (19). Type I is the most common form and predominantly affects men (20). Fat deposits are symmetrically distributed in the upper body, including the neck, shoulders, and upper limbs (4). The typical clinical picture of a patient with type I is the slow enlargement of painless fat masses in the neck, often giving the appearance of a “horse collar.” In some cases, disease progression is accompanied by weight loss (5). Type II occurs with equal frequency in both sexes and is not associated with excessive alcohol consumption. Fat deposits are localized mainly in the upper back, deltoid area, upper arms, buttocks, and upper thighs. In some cases, fat may also accumulate in the upper abdomen. Patients with Type II MD often present with weight gain, making this form easily mistaken for common obesity.

**Figure 1:**
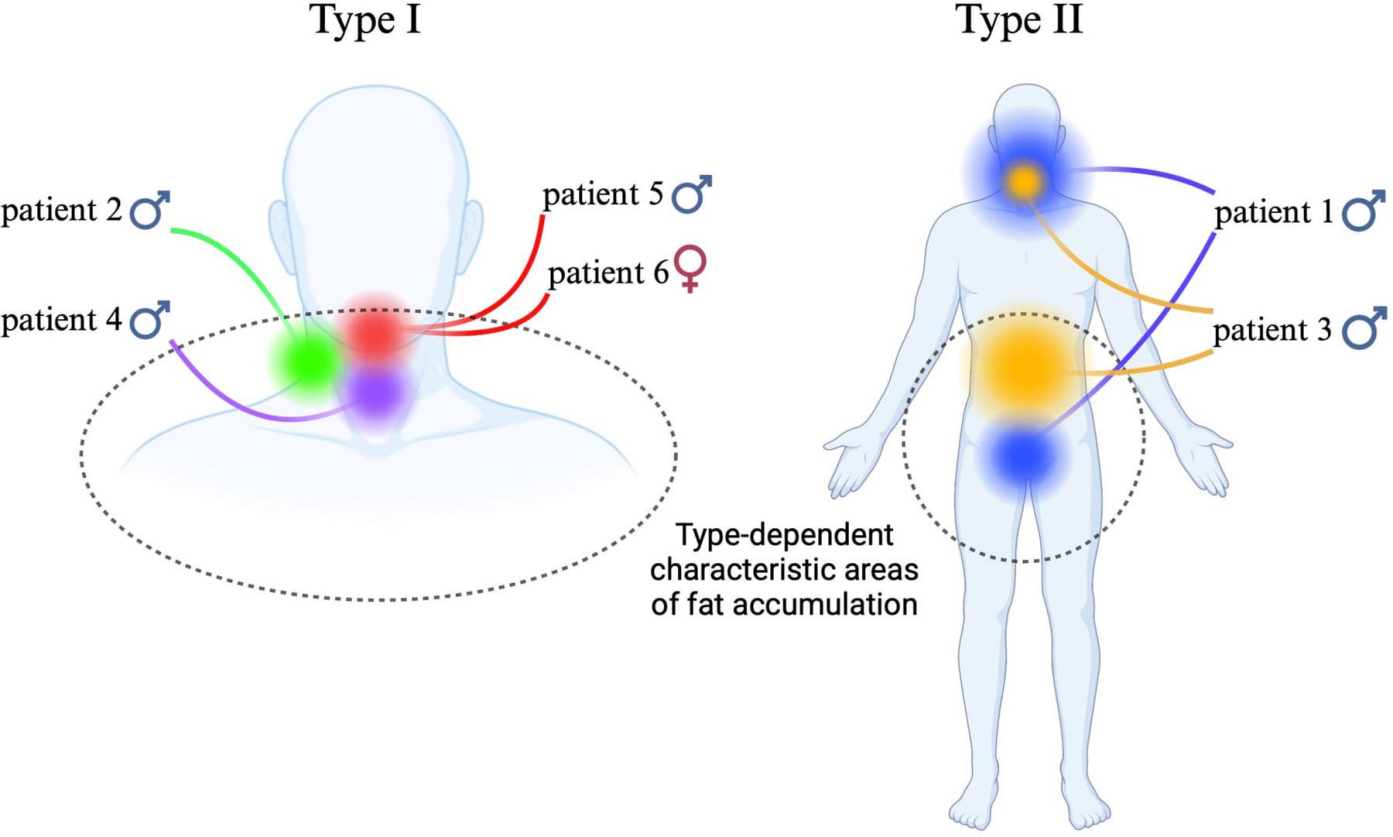
Figure illustrating the distribution of fat mass accumulation in individual patients, according to Enzi's classification. Figure 1 created with BioRender.com.

Donhauser et al. subdivided Type 1 into three distinct subtypes, based on the specific adipose tissue distribution. Type I, called neck distribution, or even more specifically “horse collar”, mirrors the classic Type I, with adipose tissue primarily located in the neck, upper trunk, and upper arms. Type II, called pseudoathletic appearance, involves fat accumulation in the upper trunk and limbs, creating a muscular, “athletic” appearance, which can be mistaken for normal obesity due to the even distribution of fat in the torso and limbs. It is also important to notice that type II does not have a gender preference, in contrast to type I. Type III, also known as gynecoid appearance is characterized by fat accumulation in the lower body, particularly in the buttocks and thighs, leading to a gynecoid body shape similar to female-pattern obesity (7). Donhauser separated type 1 into 3 subdivisions and included type 2, also known as type IV, which is a continuation of the first three types (21). Type IV is the abdominal type, with the lipomas being located, as the name suggests, in the abdomen of the patient (22). It should be emphasized that more than one distribution pattern may coexist in the same patient Figure 1.

Patients were classified into type I and type II based on the location of fat deposits. Type I had localized lesions, mainly in the neck and submandibular region, while type II exhibited more widespread deposits across multiple regions.

Although standardized diagnostic criteria for MD have not been established, diagnosis is primarily based on clinical presentation, patient history, biopsy findings, and imaging studies (CT and MRI) (23). Magnetic resonance imaging (MRI) is a preferred technique, as it provides high-resolution, detailed visualization of adipose tissue and its distribution, which is critical for accurate diagnosis. Computed tomography (CT) is also valuable, particularly for evaluating fat deposits in the thoracic and abdominal regions (24). Ultrasound, while less sensitive, can reveal symmetrical thickening of the subcutaneous fat layer, often manifesting as irregular or cord-like echoes. However, ultrasound alone is insufficient to establish a definitive diagnosis.

Accurate differential diagnosis is essential in MD. The main conditions to be considered include obesity, Cushing's syndrome, familial multiple lipomatosis, liposarcoma, and other forms of lipomatosis (7). Because these conditions may present with a similar clinical picture, radiological imaging and histopathological evaluation are required for distinction (25). Histopathology is particularly important for excluding malignancies such as liposarcoma. Although laboratory tests are not diagnostic for MD, they may assist in identifying associated metabolic abnormalities, including liver dysfunction and thyroid disorders (26).

Madelung's disease is primarily treated symptomatically, with weight reduction and alcohol elimination (1). To date, there is no effective pharmacological treatment for MD. Non-surgical methods like phosphatidylcholine, multivitamins, hyaluronic acid, yohimbine or collagenase have been reported to slow the progression of fat mass growth, although they do not reduce its volume. These approaches require repeated administration and demonstrate only limited, temporary efficacy (23). Fibrate medications, which are PPAR-αactivators, work by reducing fat accumulation through the suppression of brown adipose tissue protein expression. Additionally, a β2-adrenergic stimulant, such as salbutamol, has been employed to prevent fat buildup and enhance energy expenditure (7). Nevertheless, the effectiveness of pharmacotherapy remains unproven, and surgical intervention should be considered the mainstay of treatment in all cases.

MD is closely associated with metabolic dysfunction, mitochondrial abnormalities, and alcohol-related injury. Surgical treatment removes fat masses but does not address underlying metabolic disturbances. Therefore, adjuvant nutritional and pharmacologic strategies may be beneficial. Mitochondria-targeted antioxidants (e.g., MitoQ, alpha-lipoic acid, L-carnitine) and polyphenols (e.g., resveratrol) have been shown to improve mitochondrial efficiency, stimulate adipose tissue browning, and reduce recurrence risk. The major challenge is poor bioavailability of bioactive compounds; approaches such as nanoemulsions and liposomal formulations may enhance absorption. Framing MD as part of a systemic metabolic disorder emphasizes the need for combined surgical and metabolic interventions (27, 28).

The primary treatment goals in MD are the restoration of function and the improvement of appearance. Surgical management consists mainly of lipectomy and liposuction. Lipectomy is generally favored over liposuction, as it enables more complete removal of adipose tissue and provides better control of adjacent tissue involvement. However, it is associated with a higher risk of complications, including infection, bleeding, and scarring (23). Despite these risks, lipectomy offers the significant advantage of a longer time to recurrence compared with liposuction. Liposuction, while less effective in the management of larger fat deposits, is a less invasive technique associated with a lower risk of complications and reduced scarring (20). The overall rate of postoperative recurrence is 63%, with 95% of patients experiencing a relapse of symptoms following liposuction (29). Consequently, long-term clinical follow-up is strongly recommended. The choice of surgical procedure should be guided by the severity of the disease, the patient's individual goals, and the surgeon's expertise. In the past, open surgery was the standard treatment for MD, but it was associated with a high complication rate reaching up to 17.8% (30). Klein's solution, developed in the late 1980s, consists of lidocaine, epinephrine, sodium bicarbonate, and 0.9% NaCl. It reduces bleeding by vasoconstriction while prolonging anesthesia (31, 32). Today, it is widely used in liposuction, phlebectomy, hair transplantation, and reconstructive surgery. Several studies confirmed its role in reducing intraoperative blood loss. For example, Lillis reported minimal hematocrit changes in 17 out of 20 patients after tumescent liposuction. Another study demonstrated completely bloodless fields in 38.5% of cases, minimal bleeding in 46.1%, and acceptable bleeding in 15.4% (33). Our own results confirm these findings, with no recurrence of resected adipose tissue.

## Methods

In a retrospective study conducted between 2018 and 2024 at the Clinic of Plastic Surgery in Gdańsk, 6 cases of Madelung's disease treatment were analyzed.

**Figure 2:**
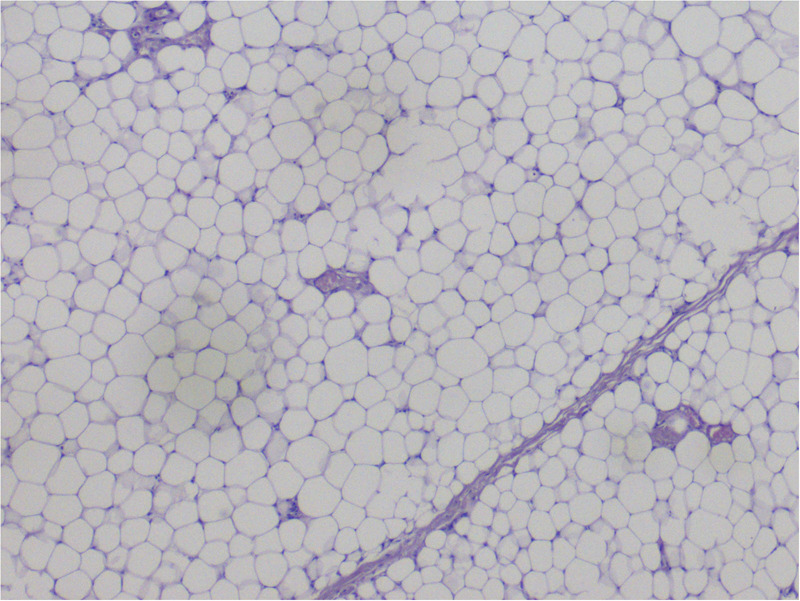
Figure presenting histopathological preparation of fat collected from a patient with madelung's disease.

All patients underwent surgical treatment involving the excision of pathological adipose tissue following a prior injection of Klein's solution Figure 2.

## Results

### Patient characteristics

**Figure 3:**
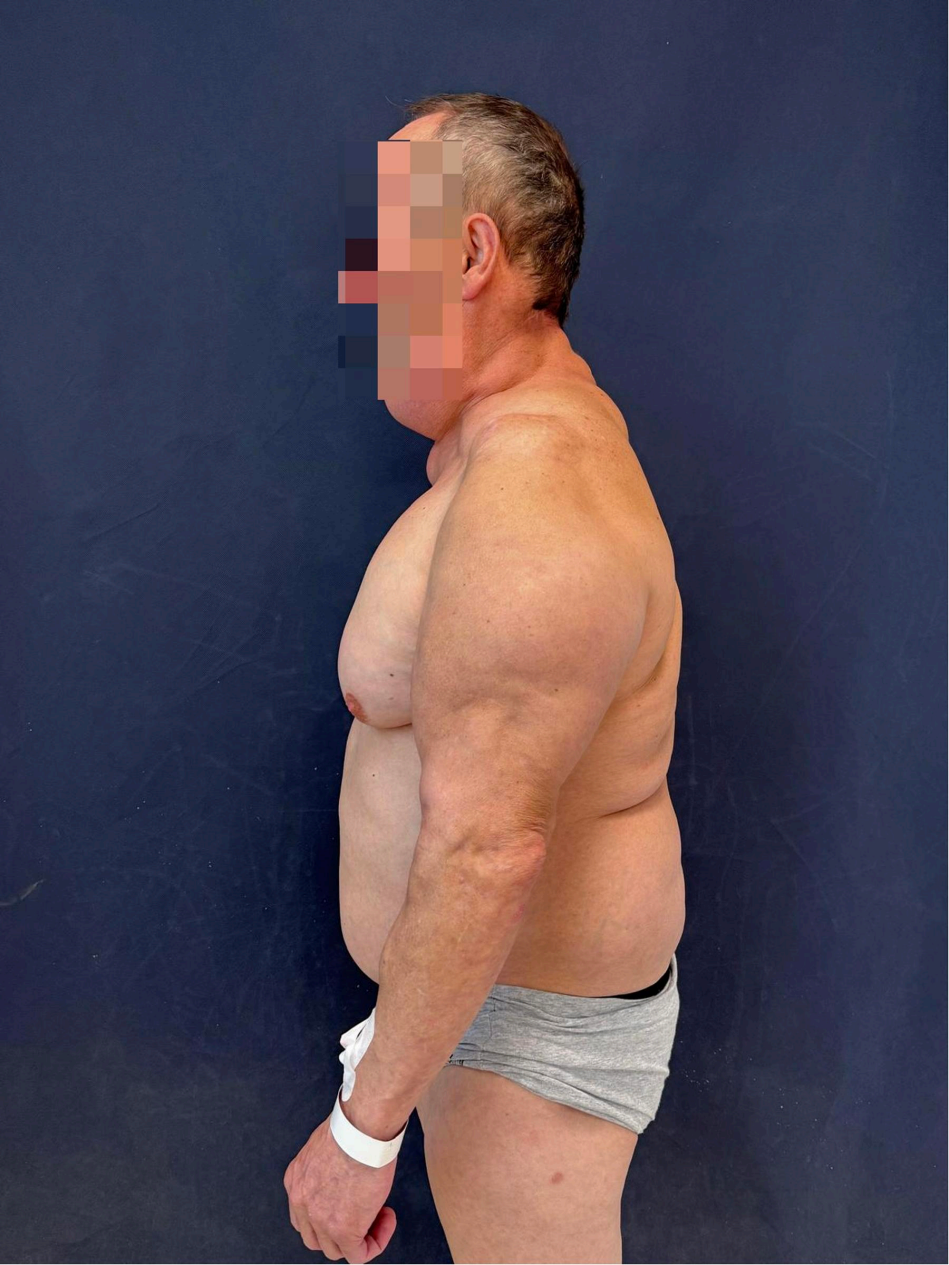
Figure illustrating one of the examined patients with a presentation of madelung's disease type 1.

- Gender: 5 males, 1 female.
- Average Age: 57 years (from 55 to 60 years old)
- Risk Behaviors:
  - Smoking: 4 patients.
  - Excessive alcohol consumption: 1 patient
- Other Health Conditions: 1 patient with gastric cancer.
- Distribution of Changes:
  - Type I (neck): 4 patients Figure 3.
  - Type II (additional abdominal and pubic area): 2 patients.
- BMI:
  - Within normal range (21): 1 patient.
  - Elevated- ranging from 26 to 33: 5 patients.

### Intervention description

All patients underwent surgical treatment involving the excision of pathological adipose tissue following a prior injection of Klein's solution. Due to the size of the masses and the tendency for intraoperative and early postoperative bleeding, which is caused by pathological proliferation of blood vessels, only one area was operated on at a time Table 1.

**Table 1:** Table presenting the intervention description of the patients.

Patient ID	Gender	Age	BMI	Other conditions	Smoking (Y/N)	Alcohol consumption (Y/N)	Allergies	Number of hospitalizations	Length of hospital stay	Lesion location	Type of change	Treatment performed	Previous hospitalization for this condition (Y/N)	Post-surgical complications	Surgical outcome	Histopathology results	Comments
Patient 1	Male	64	33	None	Yes	No	Vitamin B12	5	6	Neck and submental region	Type II	Surgical excision	No	None	No recurrence	Lipoma	None
									5	Neck and face		Surgical excision		None	No recurrence	Lipoma	None
									1	Pubic region		None		N/A	No recurrence	N/A	Infection, not eligible for surgery
									4	Pubic region		Surgical excision		Hematoma, hematoma evacuation	No recurrence	Lipoma	None
									4	Posterior cervical region		Surgical excision, liposuction (Klein Solution)		None	No recurrence	Lipoma	None
Patient 2	Male	56	26	Gastric cancer, acute pancreatitis	No	No	None	1	4	Right side of the neck	Type I	Surgical excision, end-to-end nerve repair	Yes (surgical excision of posterior cervical region lipomas)	Intraoperative injury to marginal mandibular branch of facial nerve - smile dysfunction	No recurrence	Lipoma	Awaiting another surgery
Patient 3	Male	59	30	Toxic post-alcohol liver damage	Yes	Yes	None	2	7	Abdominal wall	Type II	Surgical excision, negative pressure wound therapy (NPWT)	No	Surgical site infection (S.aureus), abscess drainage	No recurrence	Lipoma	None
									5	Submandibular region		surgical excision		None	No recurrence	Lipoma	None
Patient 4	Male	44	21	None	No	No	None	1	6	Neck and posterior cervical region	Type I	Surgical excision	No	None	No recurrence	Lipoma	None
Patient 5	Male	56	28	Metabolic disease	Yes	No	None	1	5	Neck	Type I	Surgical excision	No	None	No recurrence	Lipoma	None
Patient 6	Female	67	28	Hypertension	Yes	No	None	2	3	Submandibular region	Type I	No documentation	Yes	No documentation	No recurrence	Lipoma	None
									5			Surgical excision		Hematoma, hematoma evacuation, broad-spectrum antibiotic therapy	No recurrence	Lipoma	None

### Outcomes

The surgical technique was considered effective; no recurrences were observed.

## Discussion

According to our knowledge, this is the largest case series to date on Madelung's disease and its treatment. It is a very rare disease with an unknown etiology. Although the exact cause of Madelung's disease remains undetermined, all patients seem to align with the suspected factors contributing to its development, which include smoking, excessive alcohol consumption, or metabolic comorbidities (10). Alcohol consumption is recognized as an important risk factor in the development and progression of Madelung's disease. However, assessing this factor in clinical practice may be challenging. Alcohol use remains a sensitive and often stigmatized topic, and patients may be reluctant to disclose regular consumption due to embarrassment or social concerns. This could explain why only one of the six patients in our cohort admitted to alcohol intake, despite its established role in the pathogenesis of the disease.

Adipose tissue is not only structural but also an active endocrine organ. One of the secreted adipokines is progranulin (PRG), a pleiotropic and proangiogenic factor. PRG can independently stimulate angiogenesis and act synergistically with VEGF. These mechanisms suggest that abnormal fat growth in Madelung's disease may be biologically driven, not merely structural. This provides a molecular and mechanistic link between adipose tissue, angiogenesis, and pathological remodeling (1).

Currently, there is no possibility for causal treatment, and the only options available to patients are symptomatic treatments such as lifestyle modifications or surgical procedures (20, 35). In all cases presented, excision of the fatty tissue was performed after prior infiltration with Klein's solution, resulting in a good aesthetic effect and, over several years of observation, no recurrences in the operated areas. Although the surgical technique was found to be effective and no recurrences were observed in the analysed group of patients, several postoperative complications were noted, including haematomas, infections and temporary nerve palsy. The most common complication in the surgical treatment of Madelung's disease is undoubtedly bleeding (34). In our group of patients, we observed cases of haematomas requiring evacuation, for example in patients 4 and 6. This is related to the pathological network of blood vessels surrounding the overgrown fatty tissue. Probably, the above is related to cautious surgical treatment divided into stages-during one procedure, only one diseased area was removed. This prevented anemia during hospitalization and the necessity of potential reoperation. In all cases, a drain was left to monitor for possible bleeding.

In addition to the bleeding complications, the analysis identified other rare but significant complications. One patient developed a Staphylococcus aureus postoperative wound infection, requiring abscess drainage. Furthermore, in the same case, intraoperative damage was sustained by the marginal mandibular branch of the facial nerve, resulting in temporary smile dysfunction. This underscores the necessity for caution during procedures in areas characterised by a high concentration of nerve structures. Our findings align with the conclusions of previous studies, which have demonstrated the efficacy of the tumescent technique in reducing intraoperative blood loss (31, 33). In addition to improved safety, we observed no recurrence of resected adipose tissue, which supports the long-term durability of this method. These results highlight the value of tumescent anesthesia as a reliable adjunct in surgical management of Madelung's disease.

Reports of neurological complications directly related to surgery for Madelung's disease are extremely rare. Most data concern neuropathy as a manifestation of the disease itself. One case described brachial plexus compression caused by scar tissue after lipectomy, which required reoperation; symptoms resolved and no recurrence was observed during three years of follow-up. Overall, when performed with proper technique, surgical treatment of MD carries a very low risk of nerve injury (36). Despite the occurrence of the aforementioned complications, no long-term consequences were observed in any of the cases. This emphasizes that, with the use of appropriate surgical techniques, and in particular gradual tissue removal, the risk of serious complications can be effectively minimized, ensuring safe and satisfactory treatment outcomes for patients.

We believe that surgical treatment of Madelung's disease is effective, and its benefits for patient outweigh the potential complications. At the same time, lifestyle modifications, particularly alcohol cessation and smoking reduction, remain critical in managing the underlying risk factors and preventing disease progression. The molecular mechanisms underlying Madelung's disease remain poorly understood. The risk factors and pathogenetic hypotheses outlined in this study warrant further investigation. Given the disease's complex pathogenesis and documented correlation with metabolic issues, further molecular studies are recommended to fully understand its etiology and optimize treatment strategies.

## Conclusions

Surgical treatment of Madelung's disease in the analyzed cohort of patients showed high effectiveness in eliminating pathological fat tissue from specified body regions. Further studies are recommended to confirm these findings in a larger group of patients and to assess the long-term outcomes of treatment for this rare disease (29).

### Limitations

The study faces several limitations, primarily its small sample size (n=6) and retrospective design, which may restrict the generalizability of the findings and introduce potential selection or information biases. Given the rarity of this disease, our aim was to present all patients treated for this condition in our clinic in a retrospective study. A major limitation of the study was the unavailability of tissue samples from patients who had undergone surgery previously. Despite these limitations, the study is characterized by important strengths, including a uniform one-year follow-up for all patients, the use of a standardized surgical technique, and the absence of postoperative complications, all of which enhance the reliability of the reported outcomes.

### Suggested further research

A long-term, multicenter prospective study is recommended due to the rarity of the disease, to evaluate the efficacy and safety of this treatment method in a larger patient cohort.

## Data Availability

The data analyzed in this study is subject to the following licenses/restrictions: the data underlying this article cannot be shared publicly due to the privacy of individuals that participated in the study. The data will be shared on reasonable request to the corresponding author. Requests to access these datasets should be directed to Monika Łącka, mlacka@gumed.edu.pl.
