# Correction: Madelung's disease -a case series from a single-center experience

**DOI:** 10.3389/fsurg.2025.1727428

**Published:** 2025-11-03

**Authors:** Monika Łącka, Julia Wojciechowska, Paulina Bernecka, Hanna Szóstek, Amelia Stolp, Zuzanna Zieniewicz, Martyna Miller, Brygida Ossowska, Jerzy Jankau

**Affiliations:** 1 Plastic Surgery Department, Medical University of Gdańsk, Gdansk, Poland; 2 Student Scientific Association of Plastic Surgery, Medical University of Gdańsk, Faculty of Medicine, Gdansk, Poland; 3 Division of Embryology, Department of Anatomy, Medical University of Gdańsk, Gdansk, Poland

**Keywords:** Madelung's disease, Madelung's disease treatment, multiple symmetric lipomatosis, surgical treatment, Klein's solution, rare metabolic disorder

Affiliation 1 was erroneously given as “Plastic Surgery Department, University Clinical Center in Gdańsk, Gdansk, Poland”. Affiliation 2 was erroneously given as “Faculty of Medicine, Medical University of Gdańsk, Gdansk, Poland”.

The original version of this article has been updated.

